# Endoscopic Stricturotomy Is an Efficacious Option for Management of Strictures in Patients With Inflammatory Bowel Disease

**DOI:** 10.1093/crocol/otaa069

**Published:** 2020-10-30

**Authors:** Nabeeha Mohy-ud-din, Gursimran S Kochhar

**Affiliations:** 1 Medicine Institute, Allegheny Health Network, Pittsburgh, Pennsylvania, USA; 2 Division of Gastroenterology, Hepatology and Nutrition, Allegheny Health Network, Pittsburgh, Pennsylvania, USA

**Keywords:** inflammatory bowel disease, strictures, endoscopic balloon dilatation

## Abstract

**Background:**

Strictures are a common complication for patients with inflammatory bowel disease. Endoscopic stricturotomy (ESt) is a novel procedure for treatment of these strictures.

**Methods:**

A chart review was performed for patients with strictures who underwent ESt.

**Results:**

Eleven patients were included in the study and the total number of strictures treated was 12. The mean length of the strictures was 10.25 ± 4.36 mm. Technical success was achieved in 92% (n = 11) of the procedures. Postprocedural bleeding occurred in 9% (n = 1) of patients, and none of the patients had complications of infection or perforation.

**Conclusions:**

ESt is a safe technique with high technical success rate.

## INTRODUCTION

In patients with Crohn disease (CD), one-third of the population develops strictures within 10 years of disease onset.^1^ Chronic and severe inflammation in CD drives excessive production of extracellular matrix components such as collagen and fibronectin. Over time, this can lead to narrowing of intestinal lumen, stenosis and can eventually lead to an obstruction. Strictures are commonly located in the terminal ileum and colon, followed by strictures exclusively in the ileum, colon and less commonly in the upper gastrointestinal tract.^2^ The incidence of an anastomotic stricture after colorectal surgery can be as high as 30%.^3^

The gold standard in management of strictures, primary or secondary has traditionally been surgical. It is estimated that up to 80% of patients with CD will require at least 1 surgical resection within 10 years of diagnosis.^4^ Surgical management of strictures is efficacious, however, is associated with many risks, including intraoperative risks such as bleeding, infection, and perforation and postoperative complications such as short gut syndrome and stricture recurrence at anastomotic sites.^1^ The complication rate of surgical management of strictures in some studies has been reported be as high as 31.9%.^5^ There is always a search to find less invasive options for management of strictures.

Among various endoscopic therapies, endoscopic balloon dilatation (EBD) has been the mainstay for treatment of strictures. A meta-analysis involving 1089 patients (2664 EBDs) noted technical success of EBD was greater than symptomatic improvement in patients (92% vs 70%, respectively) and at 5 years 80% of patients had required recurrent dilatations, and/or surgery (75%).^6^ Endoscopic stricturotomy (ESt) is one such modality that has been shown recently to be effective and safe for management of inflammatory bowel disease (IBD)-associated strictures. Most of the evidence on its use has been limited to a single endoscopist from a single center in the United States. The aim of our study was to evaluate the safety and efficacy of ESt for anastomotic strictures in patients with IBD.

## METHODS

### Study Design

A retrospective chart review was performed for patients who underwent ESt at our tertiary care center between August 2018 and April 2020. The study received an Institutional Review Board (IRB) no purview determination (exemption).

### Inclusion Criteria

All adult patients (>18 years of age) with a history of IBD who underwent ESt at our tertiary care center were included.

### Exclusion Criteria

Pregnant patients and patients with strictures not related to IBD were also excluded.

### Data Collection

Patient details including age, gender, ethnicity, body mass index, age at diagnosis of CD, smoking history, comorbidities, surgical history, family history of IBD and cancers, extraintestinal manifestations of the disease, current medication use, and main symptoms (if present) related to the strictures were recorded.

Details relating to the stricture including traversability prior to the procedure, length of the stricture, location of the stricture, need for prior EBD, need for combined EBD and ESt during the index procedure, and need for recurrent procedures (EBD, ESt, and/or surgical interventions) were recorded.

Technical success was defined as immediate traversability of the scope without resistance after performance of the procedure. Data were also recorded for the time to complete procedure, and complications such as bleeding, infection, and perforation were recorded, for up to 28 days postprocedure.

### Outcomes

#### Primary outcome

The primary outcome was “*technical success*” of the procedure, defined as immediate traversability of the scope without resistance after performance of the procedure.

#### Secondary outcomes

The secondary outcomes of the study are listed as below:

##### Symptom improvement

This was defined as documentation postprocedure at a follow-up visit of improvement in patient’s symptomatology for which the procedure was performed based on patient’s subjective description of their symptoms.

##### Need for additional procedures

This was defined as need for recurrent ESt, and/or EBD and/or surgical intervention for the stricture that had been treated in the index procedure.

##### Hospitalization

This was defined as admission to hospital for a procedure-related factor such as bleeding, infection, or perforation for up to 7 days after the procedure.

##### Complications

These were defined as:

1. Clinically significant bleeding during the procedure, or for 28 days after the procedure.
2. Infection, which was defined as documentation of fever with associated symptoms, positive blood cultures, abdominal imaging noting an infectious source related to the procedure or need for antibiotics related to the procedure.
3. Perforation which was defined as perforation of an intra-abdominal organ caused by the procedure.

### Statistical Analysis

Descriptive analysis was used for all variables. Quantitative variables with a normal distribution were described as mean ± SD.

## PROCEDURAL TECHNIQUE

**Figure 1.**
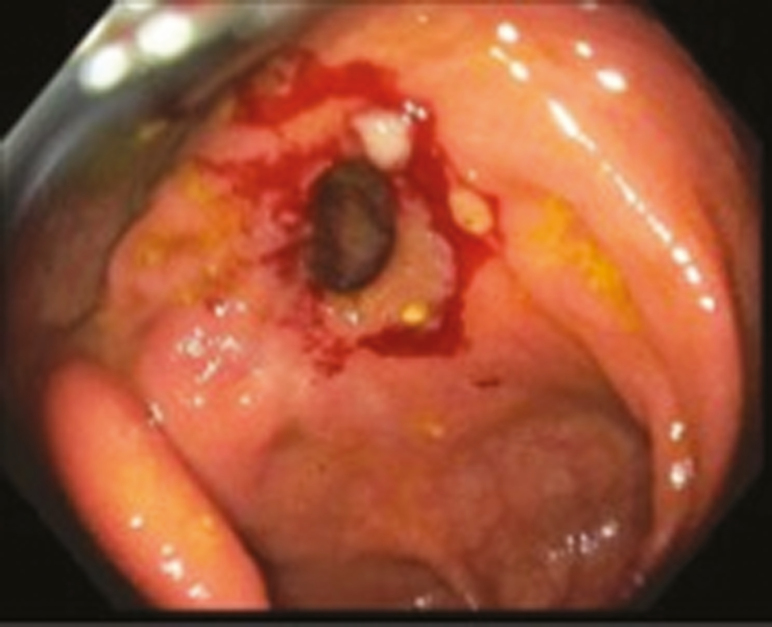
Endoscopic imaging revealing an intrinsic severe stenosis at the colonic anastomosis site.

**Figure 2.**
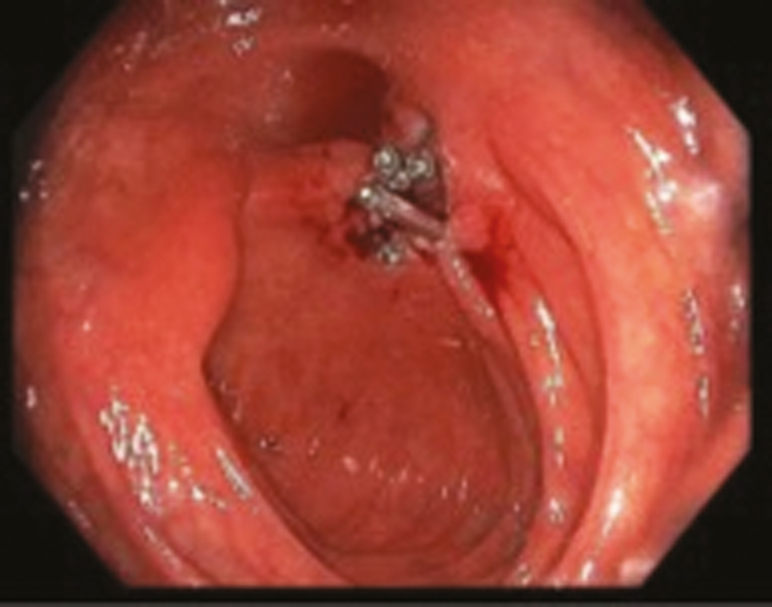
Endoscopic imaging immediately post Est, with clip closure of incised edges.

**Figure 3.**
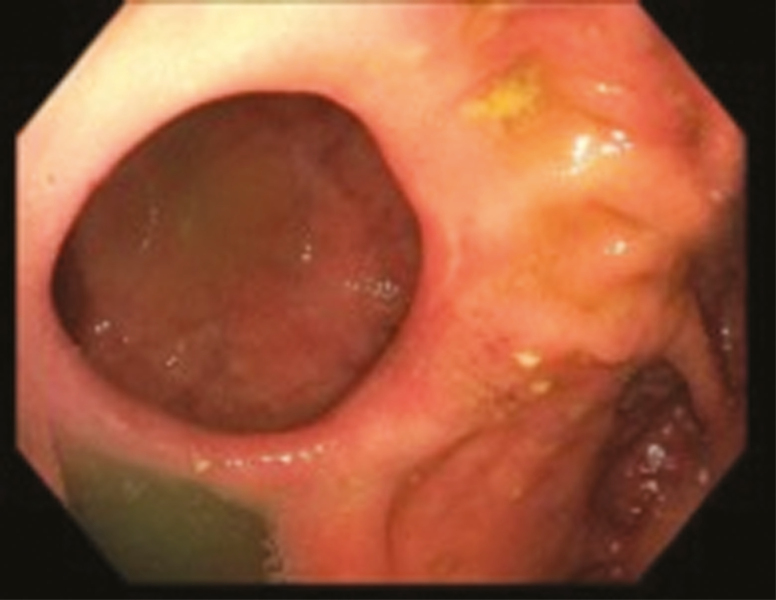
Endoscopic imaging revealing complete patency of the stricture site 1 year after performance of the ESt.

ESt is performed by a single endoscopist (G.K.) at our interventional IBD unit. All patients had preprocedure imaging (computed tomography enterography or magnetic resonance enterography). Patients underwent standard bowel preparation in anticipation of the procedure. All procedures were done under propofol sedation, administered by an anesthesiologist. After the stricture is visualized (Fig. 1), the strictures are cut in either radial, horizontal, semicircumferential, or circumferential fashion using endoscopic Nano knife (Olympus Medical Systems, Tokyo, Japan) with the current setting Endocut-I (ERBE USA, Marietta, GA). The poststricturotomy site is then visualized to assess for patency of the lumen and for any immediate complications (Fig. 2). Through the scope clips are then deployed at the site of incision in circumferential fashion. These clips serve 2 purposes: they help in prevention of the incised edges from coalescing back together, hence preventing restricture formation and also help prevent any delayed bleeding, which can happen due to the incised edges. Figure 3 is an endoscopic image revealing complete patency of the stricture site 1 year after performance of ESt.

## RESULTS

### Patient Characteristics

A total of 11 patients met the inclusion criteria and were included in the study. Sixty-four percent (n = 7) were women and the mean age of the cohort at the time of diagnosis of IBD was 31.18 ± 14.19 years, and the mean age at the time of stricturotomy was 51.45 ± 16.4 years. Approximately 36% (n = 4) of patients had an initial diagnosis of ulcerative colitis and now had an ileal pouch anal anastomosis, 18% (n = 2) had a diagnosis of Crohn colitis, and 46% (n = 5) had a diagnosis of CD of the small bowel and colon.

Ninety-one percent (n = 10) of patients had undergone bowel-related surgery before the ESt. Baseline characteristics of patients are listed in Table 1. At the time of the ESt, 36% (n = 4) patients were on vedolizumab therapy, 18% (n = 2) adalimumab, 9% (n = 1) infliximab, 9% (n = 1) ustekinumab, and 27% (n = 3) were on no therapy for their IBD. None of the patients in our study were on steroids at the time of ESt. The mean baseline weight of patient’s undergoing the stricturotomy was 71.99 ± 16.69 kg. Eighteen percent (n = 2) were active smokers, 55% (n = 6) had never smoked, and 27% (n = 3) were past smokers. Forty-five percent (n = 5) had a family history of IBD and 27% (n = 3) had a family history of colorectal cancer. Nine percent (n = 1) had a concurrent diagnosis of primary sclerosing cholangitis. The most common presenting complaints of patients were as follows: 72% (n = 8) presented with abdominal pain and bloating, 9% (n = 1) presented with complaints of bloating and nausea, and 18% (n = 2) presented with complaints of excessive straining while defecating. None of the patients included in our study had extraintestinal manifestations of IBD. About 91% (n = 10) patients had improvements in their symptoms after performance of ESt, which was assessed in a follow-up clinic visit.

**Table 1.** Baseline Characteristics of Patients

Mean age at diagnosis of IBD (y ± SD)	31.18 ± 14 y
Mean age at time of stricturotomy (y ± SD)	51.45 ± 16.4 y
Females, n (%)	7 (64)
Males, n (%)	4 (36)
Symptoms prior to stricturotomy	
Abdominal pain and bloating, n (%)	8 (72)
Bloating and nausea, n (%)	1 (9)
Straining while defecating, n (%)	2 (18)
Mean weight at time of stricturotomy (kg)	71.99 ± 16.69 kg
Mean length of stricture (mm)	10.25 ± 4.36 mm
Neobowel configuration of patients	
Ileocolonic anastomosis, n (%)	6 (55)
J-pouch, n (%)	4 (36)
Surgery naive bowel, n (%)	1 (9)
IBD medications at the time of stricturotomy, n (%)	
Vedolizumab, n (%)	4 (36)
Infliximab, n (%)	1 (9)
Adalimumab, n (%)	2 (18)
Ustekinumab, n (%)	1 (9)
No therapy for IBD, n (%)	3 (27)
Smoking history	
Current smokers, n (%)	2 (18)
Former smokers, n (%)	3 (27)
Never smokers, n (%)	6 (55)
Personal history of PSC, n (%)	1 (9)
Family history of IBD, n (%)	5 (45)
Family history of CRC, n (%)	3 (27)

CRC = colo-rectal cancer, PSC = primary sclerosing cholangitis.

### Stricture Characteristics

A total of 12 strictures were treated with ESt. The anatomic location of the strictures is outlined in Table 2. The median time to development of strictures after the surgery was 6 years (interquartile range 3–14). The mean length of the stricture was 10.25 ± 4.36 mm, 92% (n = 11) of the strictures were nontraversable prior to the procedure. Seventeen percent (n = 2) of the strictures had undergone dilation with EBD prior to the procedure in a different session. Thirty-three percent (n = 4) of strictures required EBD and ESt in the same session. There was a need to perform EBD after ESt in these patients as a complete circumferential cut was unable to be obtained with ESt alone. Eight percent (n = 1) required further ESt for the same stricture in a different session.

**Table 2.** Anatomic Location of Strictures

Location of Stricture	No. Strictures, n (%)
Ileocolonic anastomosis	6 (50)
J-pouch (inlet)	2 (17)
Anus (anal canal)	2 (17)
Rectal cuff	1 (8)
Terminal ileum	1 (8)

### Procedure Characteristics

The technical success rate was 92% (n = 11). The mean time to complete the procedure was 36.69 ± 18 min. Nine percent (n = 1) of the patients had bleeding post ESt which required admission to the hospital, however, the patient did not require any blood transfusions or further procedures, as the bleeding was self-limiting. None of the patients in our study had any other complications such as perforation or infection. Nine percent (n = 1) patients had excision of their J-pouch after the ESt due to pouch failure, unrelated to stricture site. The mean time to follow-up of patients was 144 ± 105 days.

## DISCUSSION

Management of strictures is a challenging problem in patients with IBD. Till date surgery remains the definitive treatment, although repeat surgeries are inevitable. While surgery has been the gold standard, EBD has been the mainstay endoscopic therapy in management of IBD-associated strictures. Gustavsson et al performed a retrospective study of patients who underwent EBD for CD-related strictures between 1987 and 2009. Out of 776 dilations, technical success was achieved in 89% of patients and at a 5-year follow-up, 52% of patients had required no further intervention or 1 additional dilation only. 1.4% of patients had a bowel perforation, 1.0% had major bleeding requiring blood transfusion, 1.3% minor bleeding, and 1.5% had abdominal pain or fever.^7^ Few other endoscopic techniques such as endoscopic intralesional steroid injection and endoscopic stenting that have been described in literature, however data in regards to efficacy are limited. A small prospective pilot study that looked at endoscopic stenting for treatment of CD intestinal strictures concluded that the procedure is associated with a high rate of complications and migrations.^8^

Lan and Shen described a study with 85 patients (127 strictures) who were treated with ESt. They noted a 100% technical success rate and noted that at a follow-up of 0.9 years, 15.3% patients required stricture-related surgery. Adverse events occurred in 3.7% of patients (9 patients had delayed bleeding and 1 patient had a perforation requiring hospitalization).^9^ The same group also performed a study comparing ESt with EBD. A total of 185 patients were included, 21 were treated with ESt and 164 with EBD. They concluded that ESt is more effective in treating patients with CD-associated strictures with a lower risk of perforation, however, carried a higher risk of procedure associated bleeding.^10^ A study comparing ESt with ileocolonic resection was also performed by the same group (37 patients treated with ESt, compared with 147 treated with ileocolonic resection). Procedure-related adverse events were 10.2% in the ESt group compared to 31.9% in the ileocolonic resection group. The study was limited by a short follow-up, however, it documented comparable surgery free survival in the ESt group with a decreased rate of complication as compared to the ileocolonic resection group.^11^

Recently the Global Interventional Inflammatory Bowel Disease Group, provided practical guidelines for various endoscopic modalities for treatment of CD strictures.^12^ Endoscopic electroincision (ESt/strictureplasty) was recommended in patients with EBD-refractory strictures in centers where technical capabilities exist. Furthermore, it was noted that electroincision can be particularly useful for anorectal strictures. However, it was noted that ESt may carry a higher risk of bleeding due to protuberant vessels in the ulcers that are formed by electrocautery, and hence the procedure should only be performed by endoscopists with appropriate training and skill.^12^

Our study is the second such experience that adds to the existing body of evidence that ESt is a safe and effective therapy for treatment of IBD-related strictures. We had a 92% (n = 11) technical success rate. In 1 patient, the scope could not be traversed poststricturotomy due to surgical anatomy and looping of the scope, however, the ESt was felt to be adequate. We had 1 patient who developed hematochezia post procedure and although he was hospitalized, he did not require any blood transfusions, or repeat procedures to treat the complication, as the bleeding was self-limiting. Furthermore, it is noted that during a mean follow-up of 144 ± 105 days none of the patients had clinical or endoscopic recurrence. ESt for treatment of IBD-associated strictures is a newer procedure that requires technical expertise and hence limited data exist in literature. Lan and Shen reported the procedure in a series of 85 patients; a total of 127 strictures were treated with ESt. The technical success rate was 100% and in the total of 272 ESt performed during the study period, 3.7% adverse events occurred: 9 with delayed bleeding and 1 hospitalization due to perforation.^9^

**Figure 4.**
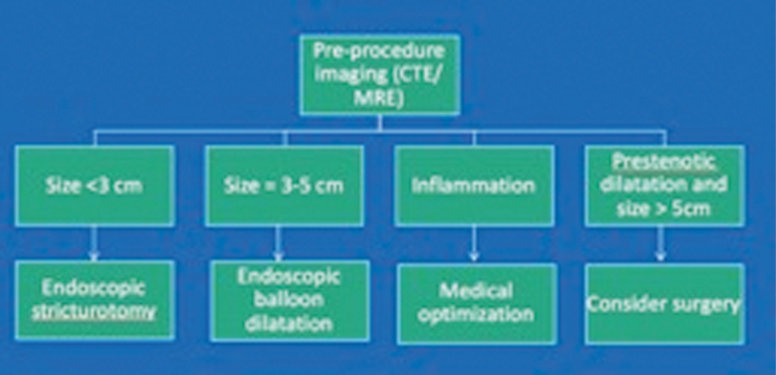
Algorithm suggesting approach to a patient with IBD-associated strictures.

EBD is considered safe and efficacious for the treatment of strictures; it also has other advantages such as preservation of bowel length, easy access to the procedure, and minimal training required to perform the procedure. However, there remains a risk of perforation with EBD. Rueda Guzmán et al quoted a 4.6% (2/45) complication rate with usage of EBD for Crohn associated intestinal strictures.^13^ In EBD, due to direct and rapid transmission of radial force, it is difficult to predict perforation. Studies have also failed to identify balloon size or duration of balloon dilatation as a risk factor for perforation. We believe with ESt, the risk of perforation is low as the endoscopist controls the cutting length and depth of strictures, although there is a concern for postprocedure bleeding with ESt. At our interventional IBD unit, we carefully select patients who can undergo ESt. Figure 4 is an algorithm of therapies chosen for management of anastomotic strictures in our unit. However, it is noted that ESt also carries its own risk of complications such as perforation and bleeding, though the risk is low as noted above. Additionally, if ESt is unsuccessful, there may be a need for surgical intervention or EBD.

Our study contributes to the much-needed literature on efficacy and safety of ESt as a treatment option for CD-associated strictures. All the previous studies that are present in literature currently show its safety and efficacy were performed at a single tertiary care center^9^ by a single endoscopist. Our study documents reproducible results at a different center performed by a different endoscopist.

The limitations of our study include it being a retrospective project and having a small sample size, thereby limiting analysis of the data. Since the first ESt at our tertiary care center was performed less than 2 years ago, our follow-up period for cases is currently limited, thereby, we cannot comprehensively assess long-term efficacy and complications of ESt currently. Relying on patients symptoms alone to assess the success of ESt also lends subjectivity to our analysis, as sometimes symptoms do not correlate with stricture severity. Heterogeneity of patient’s underlying disease conditions and history of IBD-related surgery also made it difficult to control for all confounding variables in the disease course. Additionally, patients may have undergone EBD before or simultaneously with ESt, thereby, the effect of EBD may also have been contributory to ESt’s success.

In conclusion, our retrospective case series shows that ESt for treatment of CD-associated strictures is an efficacious procedure with a high technical success rate and may be safely considered as an alternative for the management of CD-associated strictures. Further prospective studies are needed to assess its safety and efficacy compared to EBD. There is also a need for studies with a larger sample size and longer follow-up, to assess long-term success of this new therapy.

## Notes

Data Availability: Data were generated from chart review of patient’s chart after IRB approval at Allegheny Health Network. All pertinent data for the manuscript have been shared here in the manuscript.

Funding: None.

Author Contribution: Nabeeha Mohy-ud-din: Data collection, data analysis, and manuscript writing. Gursimran Kochhar: Concept, study design, performance of the procedure, preparation of manuscript, and final approval of manuscript.
